# Resilience measurement in later life: a systematic review and psychometric analysis

**DOI:** 10.1186/s12955-016-0418-6

**Published:** 2016-01-28

**Authors:** T. D. Cosco, A. Kaushal, M. Richards, D. Kuh, M. Stafford

**Affiliations:** MRC Unit for Lifelong Health and Ageing at UCL, 33 Bedford Place, London, WC1B 5JU UK

## Abstract

**Objectives:**

To systematically review and examine the psychometric properties of established resilience scales in older adults, i.e. ≥60 years.

**Methods:**

A systematic review of Scopus and Web of Science databases was undertaken using the search strategy “resilience” AND (ageing OR aging)”. Independent title/abstract and fulltext screening were undertaken, identifying original peer-reviewed English articles that conducted psychometric validation studies of resilience metrics in samples aged ≥60 years. Data on the reliability/validity of the included metrics were extracted from primary studies.

**Results:**

Five thousand five hundred nine studies were identified by the database search, 426 used resilience psychometrics, and six psychometric analysis studies were included in the final analysis. These studies conducted analyses of the Connor Davidson Resilience Scale (CD-RISC) and its shortened 10-item version (CD-RISC10), the Resilience Scale (RS) and its shortened 5- (RS-5) and 11- (RS-11) item versions, and the Brief Resilient Coping Scale (BRCS). All scales demonstrated acceptable levels of internal consistency, convergent/discriminant validity and theoretical construct validity. Factor structures for the RS, RS-11 and CD-RISC diverged from the structures in the original studies.

**Conclusion:**

The RS, RS-5, RS-11, CD-RISC, CD-RISC10 and BRCS demonstrate psychometric robustness adequate for continued use in older populations. However, results from the current study and pre-existing theoretical construct validity studies most strongly support the use of the RS, with modest and preliminary support for the CD-RISC and BRCS, respectively. Future studies assessing the validity of these metrics in older populations, particularly with respect to factor structure, would further strengthen the case for the use of these scales.

## Background

The examination of aspects of ageing beyond pathological and deficit-based models is on the rise [1–3]. Conceptual frameworks that focus on healthy ageing and resilience complement frameworks that focus on the identification and remediation of deficits, e.g. frailty [4]. Rather than merely avoiding clinical outcomes, e.g. depression, healthy ageing emphasises the high end of the functioning spectrum. Resilience involves the ability of the organism or individual to respond positively to environmental challenges (physiologically, psychologically or socially), with roots in both biomedical and psychological disciplines [5–8]

**Fig. 1:**
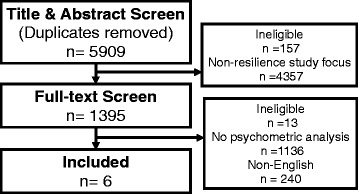
Study inclusion flowchart

Resilience features strongly in developmental psychology, examining how children positively adapt to negative circumstances, for example having injection-drug using parents, but not developing psychopathology [9]. “Bouncing back” from adversity is a fundamental principle of resilience, which has subsequently been applied beyond early-life populations to those in mid- and later-life [10]. The environmental challenges faced in early-life differ from those in later life and we know little about continuities and discontinuities in resilience across life and whether the factors that promote resilience also change. Therefore, the relevance of current models of resilience and the applicability of resilience scales should also be examined in older populations. For researchers, this information will be important in the accurate identification of variables fostering resilience. These data can then be used by clinicians to advise patients on how to increase their resilience. However, if a resilience scale is not accurately capturing resilience, any research (and subsequent clinical recommendations) resulting from the scale may provide misleading information.

A number of methodological procedures and psychometrics have been developed to capture resilience; however, according to recent reviews of resilience scales the majority of thesescales have been developed and validated in young and mid-life populations, i.e. <60 years [11, 12]. Previous studies have examined the psychometric properties of resilience scales and the theoretical underpinnings of their development by reviewing validation studies and the conceptual frameworks used in the development of these scales [11, 12]. These reviews provide insights into the validity of resilience scales in younger populations. However, these metrics should be validated in older populations. The aim of the present study is to systematically review the literature examining the reliability and validity of resilience scales that have undergone psychometric examination in older populations.

## Methods

### Search strategy

A systematic review across Scopus and Web of Science databases. Scopus is described as the largest abstract and indexing database, providing 100 % coverage Medline, Embase, and Compendex databases [13]. Web of Science is, similarly, a large abstract and indexing database, providing 100 % coverage of Science Citation, Arts & Humanities Indexes, and Social Sciences Citation databases. Searches were conducted between 05/02/2015 and 11/02/2015 using terms “resilience” AND (ageing OR aging)” for articles published on any date prior to the date of the search. . Additionally, reference lists and relevant articles were hand searched.

### Screening

Independent title/abstract and full text screening was conducted (TDC, AK, MS). Screening results were compared and disagreements concerning inclusion/exclusion were resolved via discussion.

### Inclusion criteria

Studies were included in the final analysis if they met the following criteria: i) original peer-reviewed research, ii) sample population aged ≥60 years, iii) conducted a psychometric evaluation of an existing resilience scale.

### Exclusion criteria

Studies were excluded if they met the following criteria: i) ineligible article type, i.e. conference proceeding, editorial, commentary, perspective, book chapter, book review, dissertation, or ii) published in a language other than English.

### Data extraction

Psychometric scales that had validation studies conducted with older adults were identified for extraction. Data on the psychometric robustness of the scales were collected, specifically with regards to: internal consistency, which reflects the extent to which components of a scale all measure the same construct (using Cronbach’s alpha [14]); convergent validity, which reflects the degree to which a scale’s scores align with other scales that measure similar constructs; discriminant validity, which reflects the degree to a scale’s scores diverge from other scales that measure contrasting constructs; construct validity, which reflects the degree to which a scale captures the phenomena it intends to, e.g. factor structure [15].

## Results

### Included studies

The database search returned 5909 articles (after removing duplicates) of which 426 studies had used psychometric scales. The six studies that met inclusion criteria used three different psychometrics: the Connor-Davidson Resilience Scale [16], the Wagnild & Young Resilience Scale [17], the Brief Resilient Coping Scale [18] (Fig. 1) (Table 1).

**Table 1:** Demographic characteristics of studies included in the review

Study	n	Age				Country	Female (%)	Married (%)	Population	Language
		Min	Max	Mean	SD					
Girtler, et al. (2010)^a^ [26]	178	–	–	63.9	14.6	Italy	–	–	Community–dwelling	Italian
Goins, et al. (2013)^b^ [25]	160	–	–	67.9	9.9	USA	68.8	47.8	American Indians	English
Lamond, et al. (2008) [24]^b^	1395	60	91	72.7	7.2	USA	100	–	Community-dwelling	English
Resnick & Inguito (2011)^a^ [28]	163	67	99	86.3	5.8	USA	–	–	Continuing care retirement community	English
	101	65	97	80.0	7.6	USA	–	–	Continuing care retirement community	English
Tomas, et al. (2012)^c^ [30]	133	60	84	71.7	6.9	Spain	–	66.9	Community-dwelling	Spanish
von Eisenhart Rothe, et al. (2013)^a^ [27]	3712	64	94	72.0	5.8	Germany	52.0	–	Community-dwelling	German

^a^Validation of Resilience Scale ^37^

^b^Validation of Connor-Davidson Resilience Scale (CD-RISC) ^15^

^c^Validation of Brief Resilient Coping Scale ^17^

### Included psychometrics

#### Connor-Davidson Resilience Scale

The Connor Davidson Resilience Scale (CD-RISC) is a 25-item metric developed from previous work by Kobasa [19], Rutter [7], and Lyons [20], with a theoretical grounding in stress, coping and adaptation research [12]. Items are scored on a 5-point scale (0–4) with higher scores reflecting higher levels of resilience. The scale was originally piloted with samples from the general population (*n* = 577), primary care (*n* = 139), psychiatric outpatients (*n* = 43), generalised anxiety patients (*n* = 24), and post-traumatic stress disorder patients (*n* = 22) with a mean age of 43.8 years (SD 15.4 years) [16]. In its development, five latent factors were identified: personal competence, trust/tolerance/strengthening effects of stress, acceptance of change and secure relationships, control, and spiritual influences.

The CD-RISC demonstrated adequate reliability and construct validity in its initial development. Cronbach’s alpha (a reliability metric for capturing internal consistency with cutoffs above 0.7 deemed acceptable) for the scale was 0.89 in the pilot study [16]. Subsequent reviews of studies examining the CD-RISC’s psychometric rigor in adolescent/adult samples support these initial findings [11, 12].

#### Wagnild & Young’s Resilience Scale

Items in the Resilience Scale (RS) were derived from a qualitative study of 24 older women (aged 67–92) [17, 21]. Verbatim components of these interviews were used in the pilot RS, which was conducted with 39 undergraduate nursing students [17]. All 25 items are positively scored on a 7-point scale from 1 (disagree) to 7 (agree), with possible scores ranging from 25 to 175. Higher scores indicate greater resilience. Internal consistency in the initial development of the RS was 0.89 [17]. The RS has demonstrated adequate reliability and construct validity in subsequent studies of teenagers and young adults [22, 23] as well as in a sample of adults aged 53–95 [17] (that did not meet inclusion criteria, i.e. minimum age ≥60 years, for the present study).

#### Brief Resilient Coping Scale

The Brief Resilient Coping Scale (BRCS) [18] is a four-item scale originally developed in two samples of adults with rheumatoid arthritis (*n* = 90, 140) with mean ages of 46.0 (SD 11.8) and 57.8 (SD 1.25) years. Each item is scored from 1 to 5, therefore scores range from 4 to 20 with a mean of 14.81 (SD 2.95) in the pilot study. Internal consistency for the pooled sample was 0.69. Exploratory factor analysis revealed a two factor structure [18].

### Psychometric properties in older samples

#### Connor-Davidson Resilience Scale

Two studies investigated the psychometric properties of the full CD-RISC [24, 25], additionally Goins, et al. [25] examined the abbreviated 10-item CD-RISC (CD-RISC10). Mean CD-RISC scores were 75.7 (SD 13.0) and 83.0 (SD13.4); CD-RISC10 mean score was 33.5 (SD 6.2). CD-RISC mean item correlation was 0.61 (SD 0.13) [24]. Principal components analysis revealed a four-factor structure [24] using Kaiser criterion to determine the number of factors, i.e. Eigenvalues >1. Confirmatory factor analysis suggested a uni-dimensional factor structure [25]. Significant positive correlations were observed between self-efficacy, self-mastery and social support scales while a significant negative correlation was observed with depression. Internal consistency, i.e. Cronbach’s alpha, ranged from 0.88 to 0.93 (Table 2).

**Table 2:** Psychometrics characteristics of the Connor-Davidson Resilience Scale

						Number of factors		Correlation (r)			
	Scale	n	Mean score	SD	Cronbach’s α	EFA	CFA	CES-D	GSES	PSMS	MOS-SSS
Lamond, et al. (2008) [24]	CD-RISC	1395	75.7	13.0	0.92	4	–	–	–	–	–
Goins, et al. (2013) [25]	CD-RISC	160	83.0	13.4	0.93	–	1	–0.51**	0.47**	0.29**	0.27**
	CD-RISC10		33.5	6.2	0.88	–	–	–0.51**	0.45**	0.31**	0.21*

*SD* standard deviation; *EFA* Exploratory Factor Analysis; *CFA* Confirmatory Factor Analysis; *CES-D* Center for Epidemiologic Studies Depression Scale ^42^; *GSES* General Self-Efficacy Scale ^43^; *PSMS* Personal Self-Mastery Scale ^44^; *MOS-SSS* Medical Outcomes Study Social Support Survey ^45^

*p < .01

**p < .001

#### Wagnild & Young’s Resilience Scale

The full RS was psychometrically examined in two studies [26], additionally the five- (RS-5) and 11-item (RS-11) RS were examined in a single study [27]. The Resnick & Inguito [28] study conducted RS validations in two samples (Table 1).

The full RS demonstrated a Cronbach’s alpha ranging from 0.85 to 0.91. In Resnick, et al. [28] exploratory factor analysis revealed a unidimensional model in the whole sample. Confirmatory factor analysis revealed an unacceptable fit, i.e. factor loadings below 0.50 [29], for 7 of 25 items for the unidimensional model; however, Rasch analysis indicated a fair fit to the data. Girtler, et al.’s [26] exploratory factor analysis revealed a six factor structure (meaningfulness, self-reliance, perseverance, existential aloneness, equanimity A, equanimity B) using the Kaiser criterion.

Analysis of the RS-11 revealed a Cronbach’s alpha of 0.86 and a single latent factor [27]. The RS-5 revealed a Cronbach’s alpha of 0.80. Significant positive correlations were observed with ego-resilience, (a personality trait resilience scale) and general health, as well as a significant negative correlation with depression (Table 3).

**Table 3:** Psychometrics characteristics of the Wagnild & Young Resilience Scale

				Number of factors		Correlation (r)		
	Scale	n	Cronbach’s α	EFA	CFA	ER	GHQ	BDI-II
Girtler, et al. (2010) [26]	RS	178	0.86	6	–	0.59*	0.45*	−0.31*
Resnick & Inguito (2011) [28]	RS	101	0.91	1	1	–	–	–
	RS	163	0.83		1	–	–	–
von Eisenhart Rothe, et al. (2013) [27]	RS-11	3712	0.86	1	–	–	–	–
	RS-5		0.80	–	–	–	–	–

*SD* standard deviation; *EFA* Exploratory Factor Analysis; *ER* Ego-Resilience Scale ^46^; *GHQ* General Health Questionnaire ^47^; *BDI-II* Beck Depression Inventory Second Edition ^48^

*p < .0001

#### Brief Resilient Coping Scale

A single study examined the psychometric properties of the BRCS [30]. Cronbach’s alpha was 0.83 and inter-item correlations ranged from 0.44 to 0.69. Confirmatory factor analysis supported a one-factor structure.

## Discussion

The CD-RISC, RS and BRCS (and their abbreviated versions) all attempt to capture psychological resilience. The present review reveals acceptable psychometric properties, i.e. internal consistency, convergent validity, and discriminant validity, in samples of older adults; less robust construct validity, i.e. factor structure, in the CD-RISC and RS. The CD-RISC and its shortened version (CD-RISC10) provided supporting evidence for continued use in older populations. Similarly, Wagnild & Young’s RS and its shortened versions (RS-5, RS-11) were validated in three studies with four sample groups, providing supporting evidence for the continued use of these metrics in older populations. The BRCS provided evidence for use in older populations in the few psychometric properties that were measured; however, the BRCS would benefit from further supporting evidence.

The small number of published studies conducting psychometric validations of resilience metrics in older adults, and the comparatively small sample sizes of these studies, is a notable limitation. With our intention to study only older sample groups, to ensure the validity of the metric was specific to older adults, validity studies that included older adults alongside middle –aged and younger participants were excluded. These exclusion criteria eliminated 20 validation studies from inclusion. Previous validation studies have suggested that there is no difference in the way that resilience scales capture resilience depending on gender in younger sample [31, 32]; however, included studies did not conduct any analysis of the role of gender on resilience in aging. An inherent limitation of reviewing psychometric resilience scales is the assumption that resilience is consistent across demographic and disease states, an area that has not been fully investigated in the literature to date.

In addition to being focused on older adults, studies also sampled characteristics that differed from the original samples, i.e. American community dwelling adults, by ethnicity (American Indian [24]) and language (German [27] and Italian [26]). These additional variations may have resulted in unexpected differences in the psychometric properties of the scales, e.g. factor structure.

Previous reviews of the CD-RISC have indicated that the metric is psychometrically sound in younger populations [11]. Windle, et al.’s [11] review gave the CD-RISC the highest rating for psychometric soundness in a recent critique of resilience metrics, noting its high reliability, i.e. Cronbach’s alpha and high construct validity, i.e. theory underpinning the scale [11]. The present study supports these findings in the full CD-RISC. The internal consistency in the reviewed studies (α = 0.92, 0.93) was slightly higher than the original study (α = 0.89), which was conducted with middle-aged individuals [16]. Differences in mean scores were relatively small given the size of the confidence intervals and would likely have no clinical significance [24]. Further, convergent and discriminant validity of the scale aligns with previous validations in younger populations, i.e. significant positive correlations with self-efficacy, self-mastery, and medical outcome social support surveys and a significant negative correlation with depression [33, 34].

Despite the high levels of theoretical construct validity indicated in a review of the CD-RISC [12], the factor structures in both Lamond et al. [24] and Goins, et al. [25] differed from the original five-factor structure [16]. This may suggest that the process of resilience differs somewhat in younger cohorts; however, the CD-RISC also failed to reproduce the original five-factor structure in samples in a range of age groups, e.g. American college students [35], military veterans [36], Chinese adults [37], and Australian adolescents/ adults [38]. Given the diversity of adversity experienced throughout life and the variety of positive adaptations to these adverse events, these structural differences may be a result of resilience manifesting itself in different forms at different ages. Challenges faced in adolescence, mid-life and older age are vastly disparate, which may affect the way resilience is manifested; however, a study by Liu, et al. [31] suggests structural invariance in the CD-RISC across life. These differences in factor structure may also be due to underlying differences in the overt manifestation of resilience, or due to cultural, regional or cohort differences. Alternatively, this could be a methodological artefact; the use of Kaiser criterion as the means with which to determine the number of factors to extract has been viewed as an ineffective method [39].

The abbreviated CD-RISC10 performed better than the full version. Despite having slightly lower (although adequate) internal consistency (0.93 vs. 0.83), than the full version, the CD-RISC10 demonstrated a more stable high-order single factor structure [25]. The Goins, et al. [25] study is the first to examine the psychometric properties of the CD-RISC10 in older adults, but these encouraging results suggest that further investigation and continued use is prudent.

The RS is the most widely validated resilience metric, demonstrating appropriate psychometric properties across life in previous reviews [11, 12, 40]; these results are supported by the present review of older samples. The RS was examined in three samples across two studies, demonstrating adequate levels of internal consistency and convergent and discriminant validity in line with previous psychometric analyses in younger samples [26, 41]. The factor structure of the RS varied between studies, which may indicate that the RS quantifies resilience differently in different age groups; an important limitation to be noted by researchers. These studies did, however, also vary from the original RS development study with respect to country and language, which may have obscured the underlying reason for this latent structure inconsistency. Previous studies have also failed to reproduce the original factor structure, e.g. in Dutch [42], Russian [43] and Japanese [44] samples. The RS was developed a priori with a sample of older adults and, therefore, has strong theoretical construct validity [11, 12, 40], which suggests the factor structure should be consistent. The RS was piloted with undergraduate nurses; however, the items included in the RS were taken directly from qualitative interviews with older women [21], indicating a high level of theoretical construct validity. Although findings in the present study reflect an inconsistent factor structure, the theoretical underpinnings suggest that the RS is the most psychometrically robust resilience scale for use with older adults.

The BRCS provided adequate psychometric robustness in the few validation measures collected. The brevity of the BRCS and its ease of administration is also an advantageous attribute for researchers and clinicians. Evidence for the validity of the BRCS has been provided in a few studies; however, more psychometric research is needed, e.g. further examination of factor structure, convergent validity, divergent validity, internal consistency, in order to conclusively establish the BRCS as an effective means of capturing resilience in older adults.

## Conclusions

The CD-RISC, CD-RISC10, RS, RS-5, RS-11 and BRCS demonstrate psychometric properties that fall within acceptable ranges of internal consistency, convergent and divergent validity in older populations to warrant their continued usage; however, the factor structure of the scales was inconsistent. Amongst the three resilience scales examined, the RS has been used the most widely used and is most theoretically robust resilience scale in older samples; results from the present review suggest the RS is the most suitable resilience scale for use in older adults. The RS had the greatest number of validation studies and the strongest evidence for its use, whilst the CD-RISC provided encouraging validation studies and the BRCS preliminary evidence of its validity. Given the dearth of studies reporting the psychometric properties of resilience scales it would be prudent for prospective studies of resilience to report these data whenever a resilience scale is employed. More research will be required to further existing evidence of the utility of these resilience metrics or to develop new resilience metrics specifically for use in older populations.
